# An integrated prognostic model for diffuse large B‐cell lymphoma treated with immunochemotherapy

**DOI:** 10.1002/jha2.457

**Published:** 2022-05-03

**Authors:** Marta Rodríguez, Ruth Alonso‐Alonso, Ismael Fernández‐Miranda, Rufino Mondéjar, Laura Cereceda, Álvaro Tráscasa, Anabel Antonio‐Da Conceiçao, Jennifer Borregón, Lucía Gato, Laura Tomás‐Roca, Carmen Bárcena, Begoña Iglesias, Fina Climent, Eva González‐Barca, Francisca Inmaculada Camacho, Émpar Mayordomo, Gabriel Olmedilla, Pilar Gómez‐Prieto, Yolanda Castro, Juana Serrano‐López, Joaquín Sánchez‐García, Santiago Montes‐Moreno, Mónica García‐Cosío, Paloma Martín‐Acosta, Juan F. García, María Planelles, Cristina Quero, Mariano Provencio, Ignacio Mahíllo‐Fernández, Socorro M. Rodríguez‐Pinilla, Enrico Derenzini, Stefano Pileri, Margarita Sánchez‐Beato, Raúl Córdoba, Miguel A. Piris

**Affiliations:** ^1^ Pathology Department IIS Hospital Universitario Fundación Jiménez Díaz Madrid Spain; ^2^ Center for Biomedical Network Research on Cancer (CIBERONC) ISCIII Madrid Spain; ^3^ Lymphoma Research Group IIS Puerta de Hierro‐Segovia de Arana (IDIPHISA) Madrid Spain; ^4^ UGC Laboratorios Hospital Universitario Puerto Real Cádiz Spain; ^5^ Pathology Department Hospital Universitario Doce de Octubre Madrid Spain; ^6^ Pathology Department Hospital Álvaro Cunqueiro Vigo Spain; ^7^ Pathology Department Hospital Universitari de Bellvitge L'Hospitalet de Llobregat Barcelona Spain; ^8^ Haematology Department Institut Català d'Oncologia Hospital Duran i Reynals L'Hospitalet de Llobregat Barcelona Spain; ^9^ Pathology Department Hospital Universitario de Getafe Madrid Spain; ^10^ Pathology Department Hospital Universitario y Politécnico La Fe Valencia Spain; ^11^ Pathology Department Hospital Universitario La Paz Madrid Spain; ^12^ Haematology Department Hospital Universitario La Paz Madrid Spain; ^13^ Pathology Department Hospital Universitario Príncipe de Asturias Madrid Spain; ^14^ Experimental Hematology Laboratory IIS Hospital Universitario Fundación Jiménez Díaz Madrid Spain; ^15^ Haematology Department Hospital Universitario Reina Sofia Maimonides Biomedical Research Institute IMIBIC University of Córdoba Córdoba Spain; ^16^ Pathology Department Hospital Universitario Marqués de Valdecilla Santander Spain; ^17^ Pathology Department Hospital Universitario Ramón y Cajal Madrid Spain; ^18^ Pathology Department Hospital Universitario Puerta de Hierro Madrid Spain; ^19^ Pathology Department MD Anderson Cancer Center Madrid Spain; ^20^ Pathology Department Hospital General Universitario de Alicante Alicante Spain; ^21^ Clinical Oncology Department Hospital General Universitario Virgen de la Victoria Complejo Hospital Costa del Sol Marbella (Málaga) Spain; ^22^ Clinical Oncology Department IIS Puerta de Hierro‐Segovia de Arana (IDIPHISA) Madrid Spain; ^23^ Department of Epidemiology IIS Hospital Universitario Fundación Jiménez Díaz Madrid Spain; ^24^ Divisions of Haemato‐Oncology and Haematopathology IEO European Institute of Oncology IRCCS Milan Italy; ^25^ Haematology Department IIS Hospital Universitario Fundación Jiménez Díaz Madrid Spain

**Keywords:** DLBCL, gene expression, immunochemotherapy, diffuse large B‐cell lymphoma, prognosis

## Abstract

Diffuse large B‐cell lymphoma (DLBCL), the most frequent non‐Hodgkin's lymphoma subtype, is characterized by strong biological, morphological, and clinical heterogeneity, but patients are treated with immunochemotherapy in a relatively homogeneous way. Here, we have used a customized NanoString platform to analyze a series of 197 homogeneously treated DLBCL cases. The platform includes the most relevant genes or signatures known to be useful for predicting response to R‐CHOP (Rituximab, Cyclophosphamide, Doxorubicin, Vincristine, and Prednisone) in DLBCL cases. We generated a risk score that combines the International Prognostic Index with cell of origin and double expression of *MYC/BCL2*, and stratified the series into three groups, yielding hazard ratios from 0.15 to 5.49 for overall survival, and from 0.17 to 5.04 for progression‐free survival. Group differences were highly significant (*p* < 0.0001), and the scoring system was applicable to younger patients (<60 years of age) and patients with advanced or localized stages of the disease. Results were validated in an independent dataset from 166 DLBCL patients treated in two distinct clinical trials. This risk score combines clinical and biological data in a model that can be used to integrate biological variables into the prognostic models for DLBCL cases.

## 1 INTRODUCTION

Diffuse large B‐cell lymphoma (DLBCL) is the most frequent non‐Hodgkin lymphoma subtype. It is a highly clinically, morphologically, and biologically heterogeneous group of aggressive lymphoproliferative disorders [1]. Although more than 50% of DLBCL patients can be cured by currently available therapies and have prolonged survival, the clinical outcome of DLBCL is highly variable, emphasizing the importance of DLBCL subclassification, since this can lead to more effective personalized treatment [2]. Gene expression profiling (GEP) studies led to the discovery of several DLBCL molecular subtypes based on the cell‐of‐origin (COO): germinal center B‐cell‐like (GCB), activated B‐cell‐like (ABC) subtypes [3, 4], and a third subtype, termed “type 3” or “unclassifiable,” which does not express genes characteristic of either GCB‐ or ABC‐type cells. COO information has been found that explains a significant part of the DLBCL molecular heterogeneity [5, 6], but data concerning clinical applicability have been controversial [6, 7, 8, 9, 10, 11, 12]. The possibility of using COO as a predictor of response to Lenalidomide, Ibrutinib, or Bortezomib, when associated with R‐CHOP (Rituximab, Cyclophosphamide, Doxorubicin, Vincristine, and Prednisone) remains contentious [13, 14, 15]. Parallel efforts have revealed that a variety of molecular events leading to the deregulation of *MYC* and *BCL2* expression, or the simultaneous expression of both protein markers, also have a prognostic value, regardless of the COO [11, 16–22]. Most of these studies were performed using immunohistochemistry (IHC) to assess BCL2 and MYC expression, while the COO is widely determined using the NanoString platform.

Other prognostic markers have also been proposed, including Epstein–Barr virus (EBV), P53, CD5, CD30, PD‐L1, and others [23, 24, 25, 26, 27, 28, 29]. mostly using IHC or in situ hybridization (ISH) (e.g., EBER) markers.

Interactions between these markers are complex and, in many cases, have not been fully assessed. Here, we examine whether a customized NanoString panel including genes providing information about COO, *MYC/BCL2*, and a selection of genes known to predict survival in R‐CHOP‐treated DLBCL can integrate all these sources of information to create a robust and reproducible prognostic score. These results were validated in an independent DLBCL cohort.

## 2 MATERIAL AND METHODS

### 2.1 Study design and patients

A total of 197 DLBCL patients were retrospectively enrolled in the study, which was developed in collaboration with many Spanish hospitals working in adherence to the clinical protocols of the GELTAMO Group, under the supervision of the Fundación Jiménez Díaz Ethics Committee, and conducted in accordance with the Declaration of Helsinki. All participants signed an informed consent form at diagnosis. Samples were collected and clinical data were managed, following protocols guaranteeing the confidentiality of donor data. Material and data from other centers were anonymously transferred to the Lymphoma Research Laboratory (Pathology Department, IIS‐FJD) after obtaining approval from the corresponding ethics committees, in accordance with the relevant Spanish legislation (Law 14/2007 Biomedical Research, Law 3/2018 Protection of Personal Data and Guarantee of Digital Rights, and RD 1716/2011 of Biobanks). Diagnoses were made using whole sections. All cases were reviewed, and a consensus diagnosis was made by two expert hematopathologists (SMRP, MAP) based on the World Health Organization Guidelines from 2017. Double Hit/Triple Hit (DH/TH) large B‐cell lymphoma were not excluded from this study. Samples and clinical data from patients were provided by the following departments and biobanks: Pathology Department of Bellvitge Hospital and HUB‐ICO‐IDIBELL Biobank, funded by Instituto of Salud Carlos III (PT17/0015/0024); Pathology Department of Clínico Universitario of Santiago Hospital‐CHUS and HULA Biobank‐Biobanco do Complexo Hospitalario Universitario of Santiago de Compostela (PT17/0015/0002); Biobank of Virgen del Rocío Hospital (PT17/0015/0041); Valdecilla Biobank (PT17/0015/0019); and MD Anderson Biobank (PT17/0015/0008).

Tissue for biological studies was collected at the time of diagnosis before initiating an R‐CHOP/R‐CHOP‐like regimen. Clinical features (gender; Ann Arbor classification stage; International Prognostic Index (IPI); Eastern Cooperative Oncology Group Performance Status; lactate dehydrogenase; number of extranodal sites) were also recorded at the time of the initial diagnosis [30]. Clinical and IHC features, follow‐up time, and current status are summarized in Supporting information Tables S1 and S2. Primary mediastinal B‐cell lymphoma (PMBCL) cases were excluded from the analysis. For the IPI analysis, we reclassified the patients into three risk categories: low (0–1), intermediate (2–3), and high (4–5) risk.

The validation series comprised the 166 DLBCL patients retrospectively enrolled in two multicenter clinical trials (RHDS0305 and DLCL04) and treated with R‐CHOP and R‐CHOP‐like regimen [31, 32, 33]. Clinical and molecular features, follow‐up time, and current status are summarized in Supporting information Table S3.

### 2.2 Sample collection and processing

RNA was isolated from formalin‐fixed, paraffin‐embedded (FFPE) diagnostic tissue biopsies. Total RNA was extracted with an RNeasy FFPE kit (Qiagen, Hilden, Germany) according to the manufacturer's instructions. RNA quality and quantity were assessed with an RNA 6000 Nano kit (Agilent Technologies, Santa Clara, CA, USA) using the Agilent 2100 Bioanalyzer System (Agilent Technologies).

### 2.3 NanoString LST nCounter gene‐expression assay

We used the Research Use Only Version of the NanoString LST assay (a customized 27‐gene panel, listed in Supporting information Table S4) in conjunction with the nCounter Flex Analysis System (NanoString Technologies, Seattle, WA, USA) to determine the COO (Lymph2C× NanoString assay, WO 2018/231589 A1), and to measure *MYC* and *BCL2* gene expression [12].

Briefly, the probes were hybridized to 400 ng of the total RNA for 16 h at 65°C. Automated removal of excess probe and immobilization of probe–transcript complexes on a streptavidin‐coated cartridge were carried out in the nCounter Preparation Station. Gene expression values were normalized with respect to housekeeping genes. Normalized count data were log_2_‐transformed and agglomerative hierarchical clustering of gene expression was performed. The data were analyzed using nSolver Analysis Software 4.0 (NanoString Technologies).

COO was determined on paraffin‐embedded tissue using the Lymph2Cx NanoString assay, in which low and high linear predictor scores enable samples to be assigned to the GC and ABC groups, respectively. We classified each of the samples using the NanoString COO algorithm and the scores [34]. Samples that could not be confidently assigned to either group were considered “unclassified.” The COO based on IHC was identified by the Hans algorithm [35].

### 2.4 Tissue microarrays, immunostaining, and fluorescence in situ hybridization

Tissue microarrays (TMAs) were designed and constructed using two 0.6‐mm tissue cores per case, taken from archival FFPE patient tumor blocks, using reactive tonsils as control. Immunohistochemical staining was performed and assessed by an expert central pathology committee. All diagnostic immunostaining was done, following standardized protocols in whole sections. The panel of antibodies was chosen on the basis of their biological and clinical relevance to clinical classification and DLBCL pathogenesis. Positive tumor cells were scored in percentage classes of the following markers: CD10, BCL6, MUM1, P53, CD5, CD30, Ki67, PDL1, BCL2, MYC, EBV (EBER), CYCLIN D1, and CD20 (Supporting information Table S5).

Each TMA was analyzed and scored by at least two independent pathologists (SMRP, MAP), who considered either the cytoplasmic, membranous, or nuclear staining intensity, or the percentage of positive cells. The thresholds used were those recommended by the WHO classification of tumors of hematopoietic and lymphoid tissue criteria[1]: CD10, MUM1, and BCL6 were considered positive if >30% of the neoplastic cells stained positively. EBV (EBER) and Ki67 were considered positive in cases with more than 75% positive neoplastic cells. BCL2 and MYC were considered positive if more than 40% of the neoplastic cells exhibited distinct, strong staining. CD30, CD5, CD20, PD‐L1, and CYCLIN D1 were considered positive when they comprised ≥15% of the tumor cells. Expression of tumor protein P53 was scored as negative, positive (1–50%), or very positive (>50%).

Fluorescence in situ hybridization (FISH) was performed following the routine protocol at the Pathology Department of our hospital. Vysis LSI Break Apart FISH probes (Abbott Laboratories, Chicago, IL, USA) were used to analyze BCL2 (18q21.33 region), BCL6 (3q27.3 region), MYC (8q24.21 region), and IGH (14q32.33 region), in accordance with the manufacturer's instructions.

### 2.5 Survival analysis

Survival data were available for 197 patients with DLBCL from the discovery series and for 166 patients with DLBCL from the validation series. Follow‐up was calculated based on observation time by Kaplan–Meier curve analysis. Progression‐free survival (PFS) was calculated from the date of diagnosis to the date of disease progression. Overall survival (OS) was evaluated from the date of diagnosis to the date of death. The Kaplan–Meier method was used to estimate OS and PFS, and the log‐rank (Mantel–Cox) test was used to assess differences in OS and PFS between the patient groups.

### 2.6 Statistical analysis

All statistical analyses were performed using R 3.6.1 ( https://www.R‐project.org , R Foundation for Statistical Computing, Vienna, Austria) and GraphPad PRISM version 8.4.0 software (San Diego, CA, USA). Categorical variables are reported as percentages. Continuous variables are summarized as the median and range. For all analyses, values of *p* < 0.05 were considered statistically significant.

We performed a Cox proportional hazards analysis with the IPI, COO, and *MYC/BCL2* variables for OS and PFS in the discovery set, and validated the results in the validation series. This model gives an individual risk score for each sample. Taking the terciles of the whole series (N = 197) as cut‐off points, we generated three risk groups (high‐, intermediate‐, and low‐risk) with different prognoses.

Finally, to reinforce the robustness of the conclusions, we validated the prediction model in the validation set (N = 166).

## 3 RESULTS

### 3.1 General features of the DLBCL series

The clinical characteristics of the discovery series are summarized in Supporting information Tables S1 and S2. The series includes 197 cases diagnosed with DLBCL, subdivided into COO groups based on the results of GEP (COO by Lymph2Cx assay): 105 (53.3%) germinal center B‐cell (GC‐DLBCL) cases, 59 (29.9%) activated B‐cell (ABC‐DLBCL) cases, 30 (15.2%) unclassified DLBCL cases. Three cases were excluded due to the poor quality of the results obtained using the Lymph2Cx assay. All these patients were treated with R‐CHOP, following the guidelines of the GELTAMO group [36].

The median follow‐up for the whole series was 27 months (range: 1–211), and survival probability for the whole series was 76.84% at 27 months. Survival probability at 36 months was 70.16% and at 60 months it was 68.1%. The median follow‐up for the patients in the validation series was 63.8 months (range: 0.3–119). At the time of initial diagnosis, we observed that the median age of the series was 65 years (range: 17–91), and more than half were male (52.3%). At the time of initial diagnosis, this series consisted mostly of patients at advanced stages of the disease (stages III–IV: 63%).

IHC with specific antibodies, listed in Supporting information Table S5, was performed for clinical diagnosis. Immunohistochemical analyses of the MYC/BCL2, EBER (EBV), P53, and Ki67 markers were performed in all 197 samples. Double expression of BCL2 and MYC protein was observed in 19 cases, EBER (EBV) was positive in 9 cases, P53 was considered to be positive in 37 cases, and Ki‐67 was scored as positive in 102 cases. We did not find any significant differences between these variables with respect to either OS or PFS (data not shown).

### 3.2 Association of gene expression with prognostic significance in DLBCL

Univariate analysis to evaluate factors associated with outcome revealed significant differences. We found that IPI, COO, and *MYC/BCL2* double expression, as defined in the NanoString data, was significantly associated with shorter OS in the Cox univariate analyses (*p* < 0.0001, *p* = 0.018, and *p* = 0.046, respectively; Supporting information Table S6). The multivariate analysis showed that IPI, COO, and *MYC/BCL2* were independent predictors of OS (*p* < 0.0001, *p* = 0.030, and *p* = 0.048, respectively; Supporting information Table S6).

Univariate analysis showed that IPI, COO, and *MYC/BCL2* double‐expression variables were also significantly associated with a shorter PFS (*p* < 0.0001, *p* = 0.019, and *p* = 0.003, respectively; Supporting information Table S7). The Cox multivariate analysis indicated that IPI, COO, and *MYC/BCL2* had an independent relationship with PFS (*p *< 0.0001, *p* = 0.042, and *p* = 0.006, respectively; Supporting information Table S7).

**FIGURE 1:**
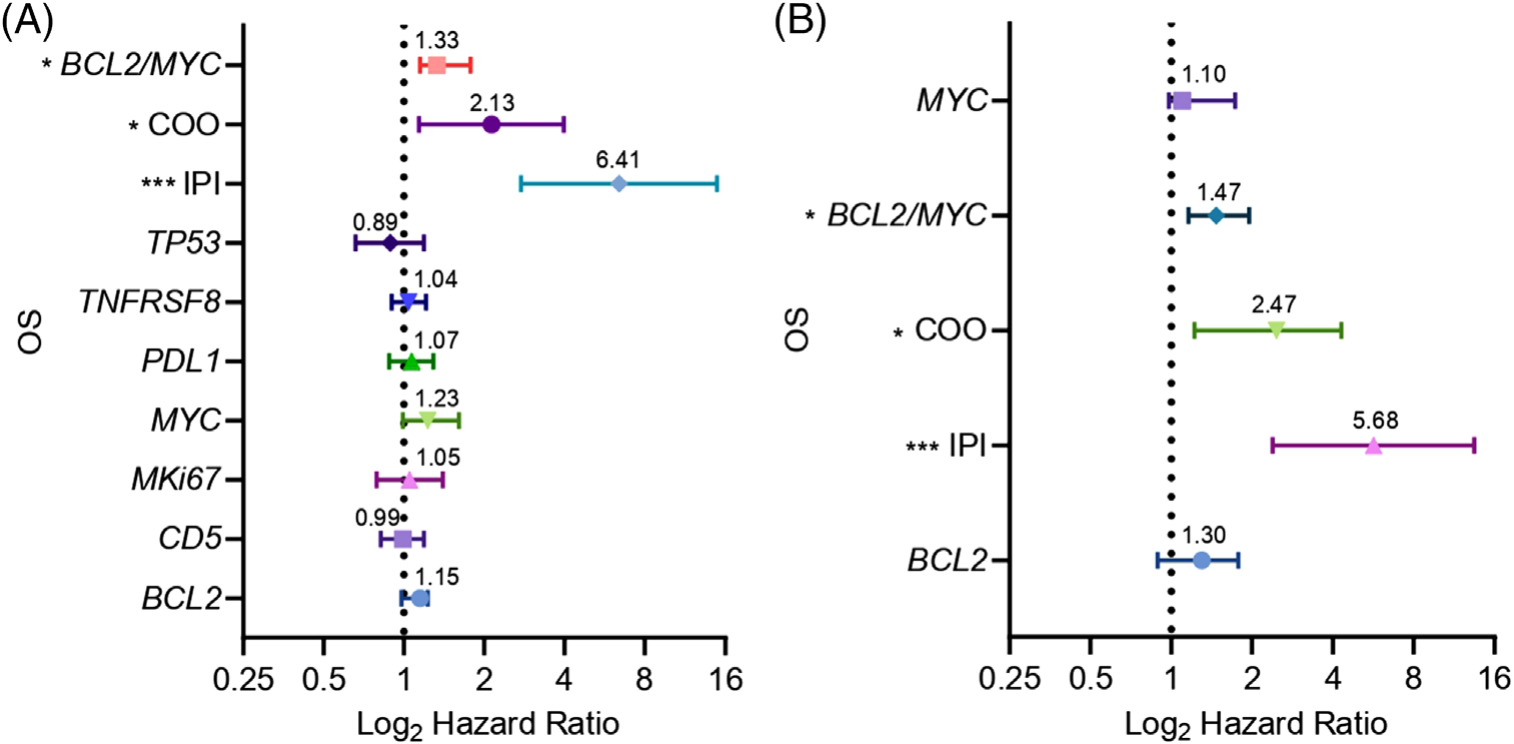
Forest plots of OS status. Hazard ratio (HR) on a log scale values from univariate and multivariate Cox analyses are represented (*p < 0.05; **p < 0.01; ***p < 0.001). Error bars show the 95% confidence interval (95% CI) of the HR. (A) Univariate analysis of these variables: *BCL2, CD5, MKi67, MYC, PDL1, TNFRSF8, TP53*, COO, and double expression of *MYC/BCL2* by NanoString gene‐expression analysis and IPI score. (B) Multivariate analysis of *BCL2, MYC*, double expression of *MYC/BCL2*, COO by NanoString gene‐expression analysis and IPI score

**FIGURE 2:**
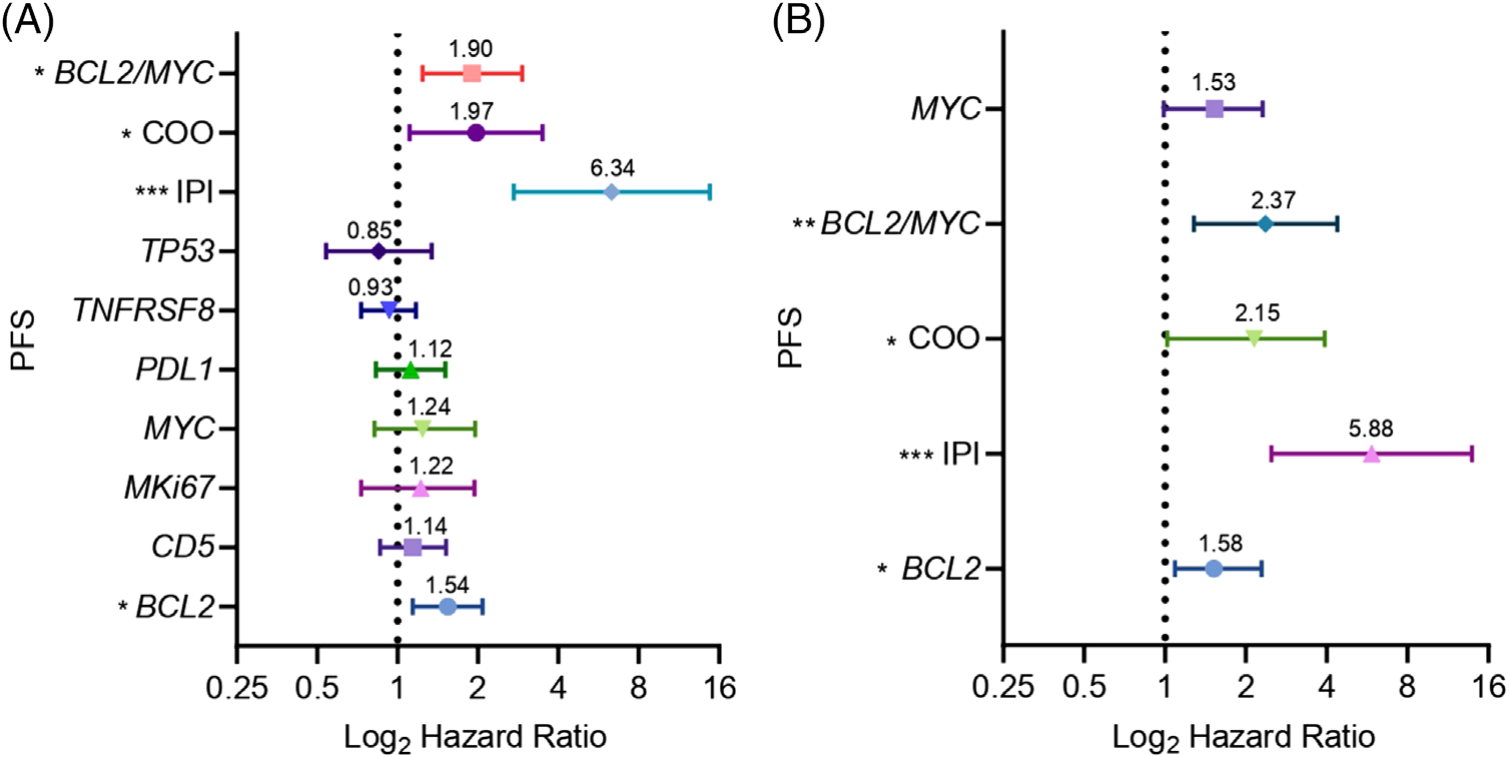
Forest plots of PFS status. Hazard ratio (HR) on a log scale values from univariate and multivariate Cox analyses are represented (*p < 0.05; **p < 0.01; ***p < 0.001). Error bars show the 95% confidence interval (95% CI) of the HR. (A) Univariate analysis of these variables: *BCL2, CD5, MKi67, MYC, PDL1, TNFRSF8, TP53*, COO, and double expression of *MYC/BCL2* by NanoString gene‐expression analysis and IPI score. (B) Multivariate analysis of *BCL2, MYC*, double expression of *MYC/BCL2*, COO by NanoString gene‐expression analysis and IPI score

Analyses to compare the effect of several risk factors on OS and PFS are illustrated by forest plots (Figures 1 and 2, respectively). The HR and 95% CI of each variable are shown in Supporting information Tables S6 and S7, respectively.

### 3.3 Prognostic model development for DLBCL risk stratification

We first assessed the prognostic value of the IPI score, COO classification, and *MYC/BCL2* double expression (NanoString platform) as individual variables in our series by Kaplan–Meier survival analysis. The IPI predicted shorter OS (*p* < 0.0001; Supporting information Figure S1A) and PFS (*p *< 0.0001; Supporting information Figure S1B) according to the Kaplan–Meier analyses, as expected. COO subtypes differed significantly in their OS probability (p = 0.0182; Supporting information Figure S2A) and showed a nonsignificant trend in the analysis of PFS (*p* = 0.095; Supporting information Figure S2B). ABC patients were found to have a worse survival probability than GC patients. In addition, *MYC/BCL2* double expression showed a trend toward significance with changes in OS probability (*p* = 0.082; Supporting information Figure S3A) and a significantly greater influence on PFS (*p* = 0.0417; Supporting information Figure S3B).

Univariate and multivariate Cox proportional hazards analyses were used to identify risk factors. Clinical and biological risk factors were evaluated, and IPI, COO, and *MYC/BCL2* were found to be significantly associated with OS and PFS (*p* < 0.05). The full results of the univariate analyses for OS and PFS are shown in Supporting information Tables S6 and S7, respectively.

We considered risk factors with a significance of *p* < 0.05 in the multivariate Cox models. Data in Supporting information Tables S6 and S7 showed that IPI, COO, and *MYC/BCL2* were independent prognostic predictors of OS and PFS, respectively. A risk score was then calculated from the final multivariate Cox regression model by incorporating the three risk factors (IPI, COO, and *MYC/BCL2*) {coxph(formula = Surv(PFS, RELAPSE = = 1) ∼ IPI + MYC‐BCL2 + NANOSTRING, data = PFS)}, which were weighted by their Cox coefficients. For the discovery series, risk scores for each patient are indicated in Supporting information Table S8.

The risk score was further split into terciles for OS (66 low‐risk, 68 intermediate‐risk, and 63 high‐risk patients) and for PFS (62 low‐risk, 67 intermediate‐risk, and 68 high‐risk patients).

**FIGURE 3:**
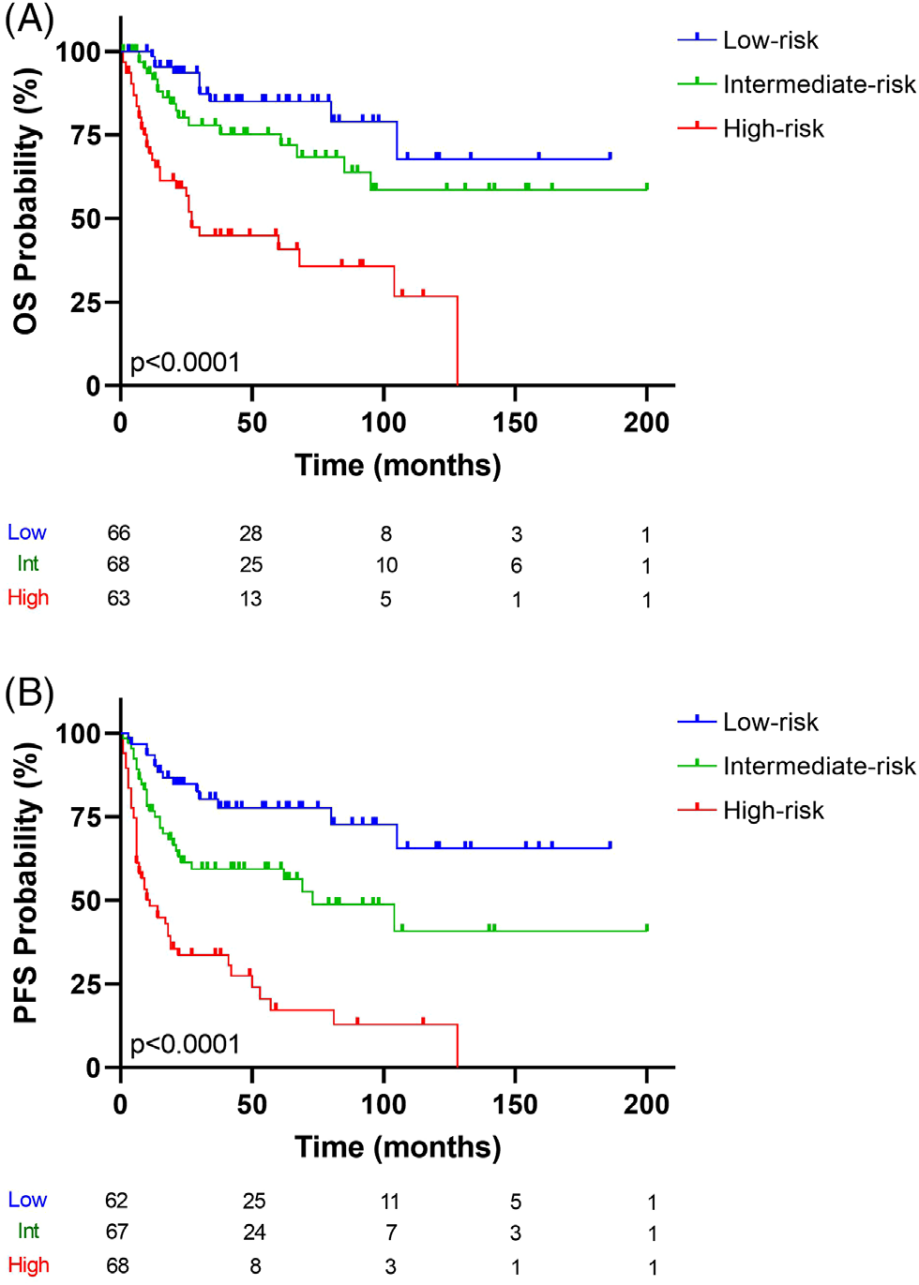
Kaplan–Meier analysis of (A) OS status and (B) PFS status. The risk‐prediction model of significant variables (IPI, COO, and double expression of *MYC/BCL2*). The discovery series was divided into three groups: blue, green, and red lines represent low‐, intermediate‐, and high‐risk, respectively. The vertical bar represents OS or PFS probability (%), while the horizontal bar indicates the follow‐up time in months. Patients at risk at the corresponding times are shown. Probabilities are those associated with a log‐rank test

A log‐rank test was also used to assess whether there were differences in survival among the groups. Figure 3A shows that the 5‐year OS of the high‐risk group was 40.7%, and that of the low‐risk group was 85.1%. Figure 3B shows that the 5‐year PFS of the high‐risk group was 17.2%, and that of the low‐risk group was 77.6%. The differences between the groups were statistically significant (low‐risk vs. intermediate‐risk vs. high‐risk group, *p* < 0.0001) (Figure 3A and B for OS and PFS, respectively).

For OS, the HRs for the three groups determined by risk score were: low‐risk, HR = 0.15, 95% CI: 0.07–0.33; intermediate‐risk, HR = 2.63, 95% CI: 1.20–5.76; and high‐risk, HR = 5.49, 95% CI: 2.54–11.90 (*p* < 0.0001).

Analysis of PFS gave the following HRs: low‐risk, HR = 0.17, 95% CI: 0.08–0.48; intermediate‐risk, HR = 3.94, 95% CI: 1.98–7.86; and high‐risk, HR = 5.04, 95% CI 2.49‐10.20 (*p* < 0.0001).

For predicting the OS and PFS probabilities, the univariate and multivariate analyses showed that the model derived by integrating the variables defining IPI, COO, and *MYC/BCL2* double expression was a better risk classifier than that obtained when these characteristics were considered separately.

As expected, ABC‐DLBCL patients were increased in the high‐risk group as compared to the intermediate‐ and low‐risk groups. On the other hand, GC samples were increased in the low‐risk group as compared to the intermediate‐ and high‐risk groups. The distribution revealed a considerable significant differences (*p* < 0.0001; Supporting information Figure S4).

DH DLBCL cases were all included within the high‐risk group when the model was applied. Survival probability of the DHL cases did not differ significantly when considered separately.

**FIGURE 4:**
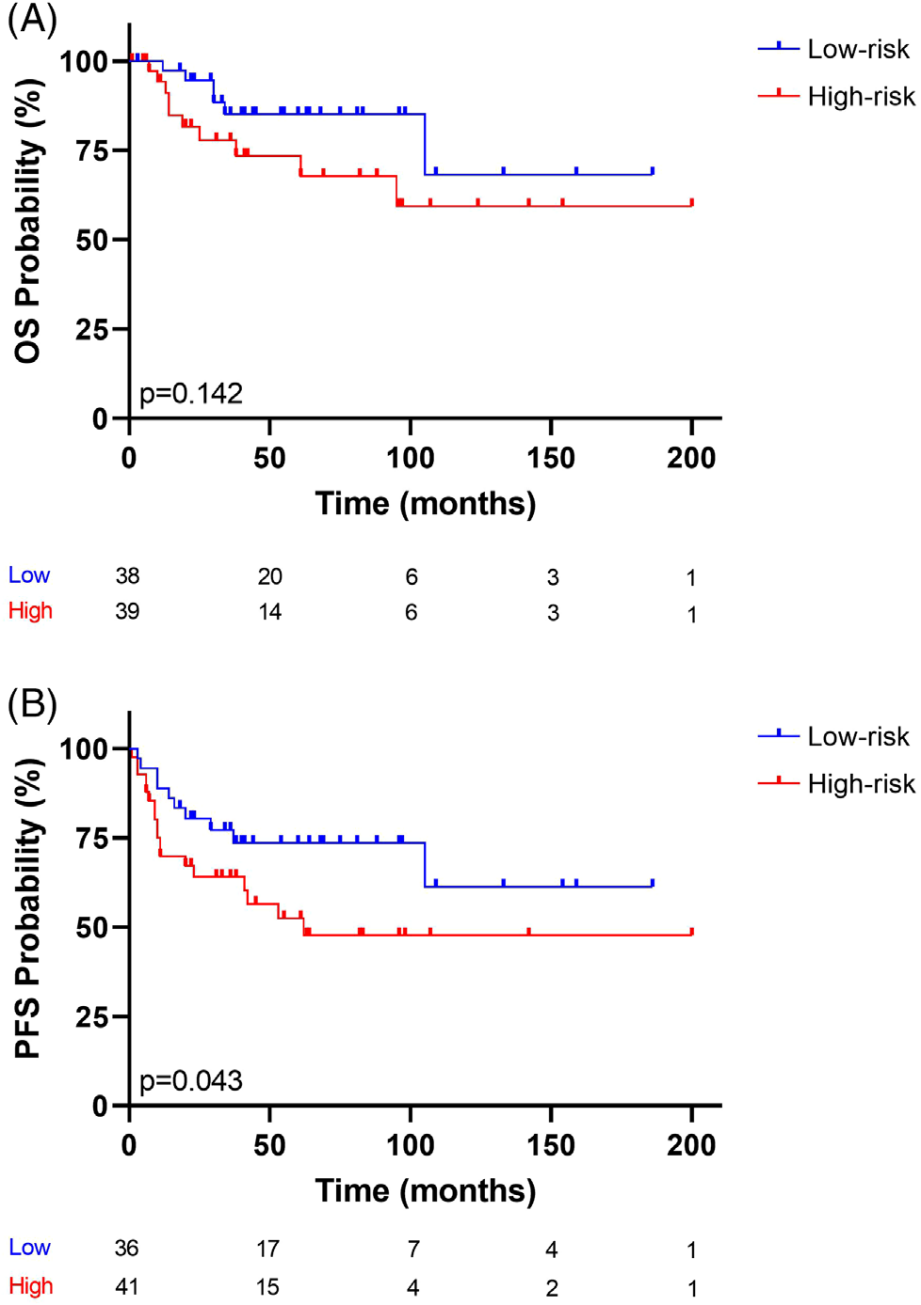
Kaplan–Meier analysis curve of (A) OS status and (B) PFS status. The risk‐prediction model was applied to patients younger than 60 years of age. Two risk groups were observed: the blue and red lines represent low‐ and high‐risk, respectively. The vertical bar represents OS or PFS probability (%), while the horizontal bar indicates the follow‐up time in months. Patients at risk at the corresponding time are shown. Probabilities are those associated with a log‐rank test

As some of the more aggressive therapeutic options may only be viable for younger patients [13], we examined whether the risk model could be used for the under 60‐year patient group (77 patients). For OS, the risk score was further split into low‐ and high‐risk groups (38 and 39 patients, respectively), using the median as the cut‐point. For PFS, low‐risk (36 patients) and high‐risk (41 patients) groups were defined in a similar way. Figure 4A shows that the 5‐year OS of the high‐risk group was 67.6%, and that of the low‐risk group was 85.1%. Figure 4B shows that the 5‐year PFS of the high‐risk group was 50.5%, and that of the low‐risk group was 73.6%. A log‐rank test found no significant differences in OS (*p* = 0.142; Figure 4A) but did reveal a significant difference in the PFS of the two risk groups (*p* = 0.043; Figure 4B).

**FIGURE 5:**
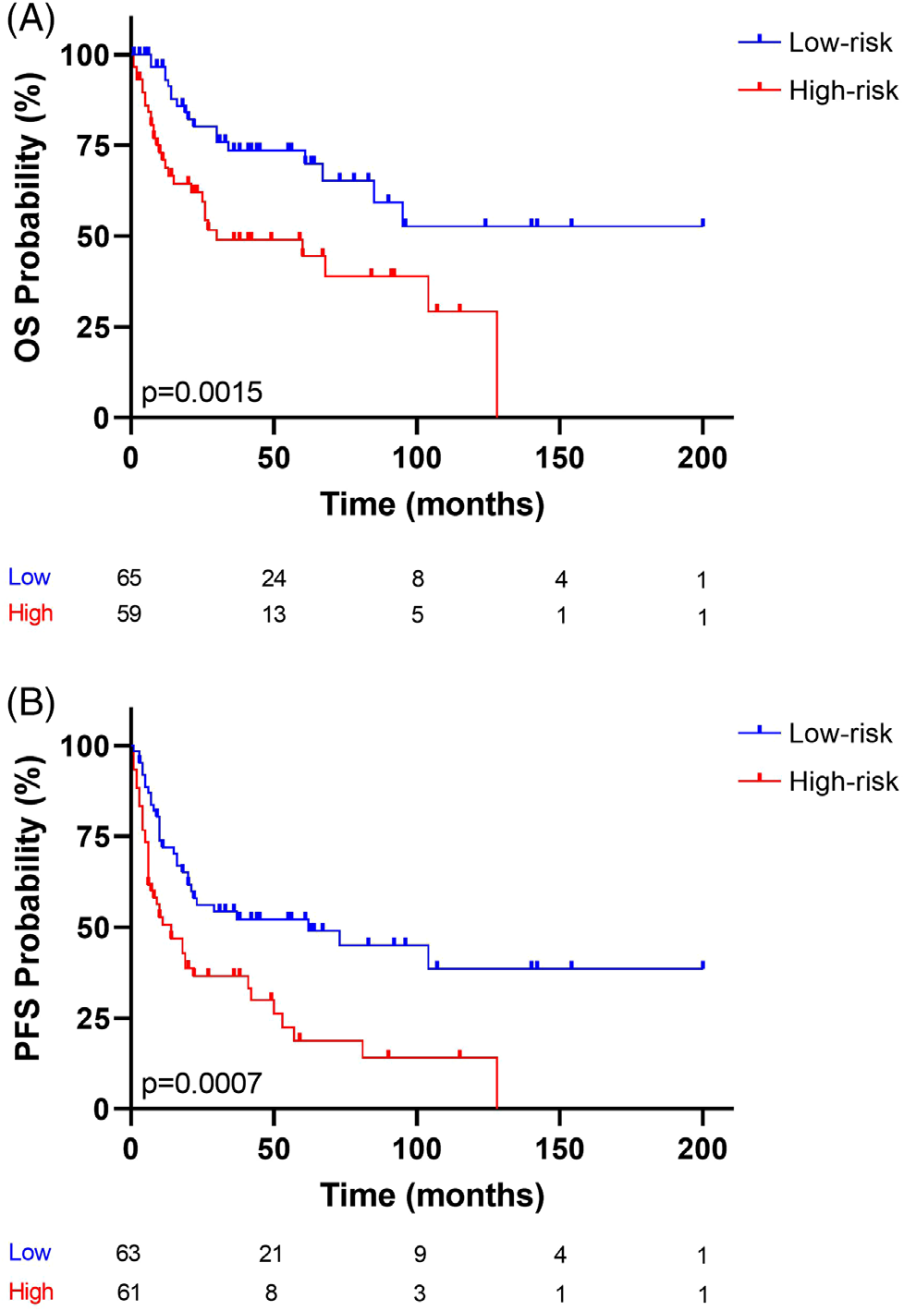
Kaplan–Meier analysis curve of (A) OS status and (B) PFS status. The risk‐prediction model was applied to patients at advanced clinical stages (stages III–IV). Two risk groups were observed: the blue and red lines represent low‐ and high‐risk, respectively. The vertical bar represents OS or PFS probability (%), while the horizontal bar indicates the follow‐up time in months. Patients at risk at the corresponding time are shown. Probabilities are those associated with a log‐rank test

We also investigated whether this risk model could be applied to advanced clinical stages (stages III–IV (124 patients), since previous studies had shown that prognostic markers and the response to therapy differs among clinical stages [37]. Therefore, for OS, the risk score was further split into low‐risk (65 patients) and high‐risk (59 patients) groups, using the median as the cut‐point. For PFS, low‐risk (63 patients) and high‐risk (61 patients) groups were compared. Figure 5A shows that the 5‐year OS of the high‐risk group was 48.9%, and that of the low‐risk group was 69.9%. Figure 5B shows that the 5‐year PFS of the high‐risk group was 17.7%, and that of the low‐risk group was 52.1%. It is noteworthy that the log‐rank test revealed significant differences in OS (*p* = 0.0015; Figure 5A) and PFS (*p* = 0.0007; Figure 5B) when restricted to cases diagnosed in advanced clinical stages.

**FIGURE 6:**
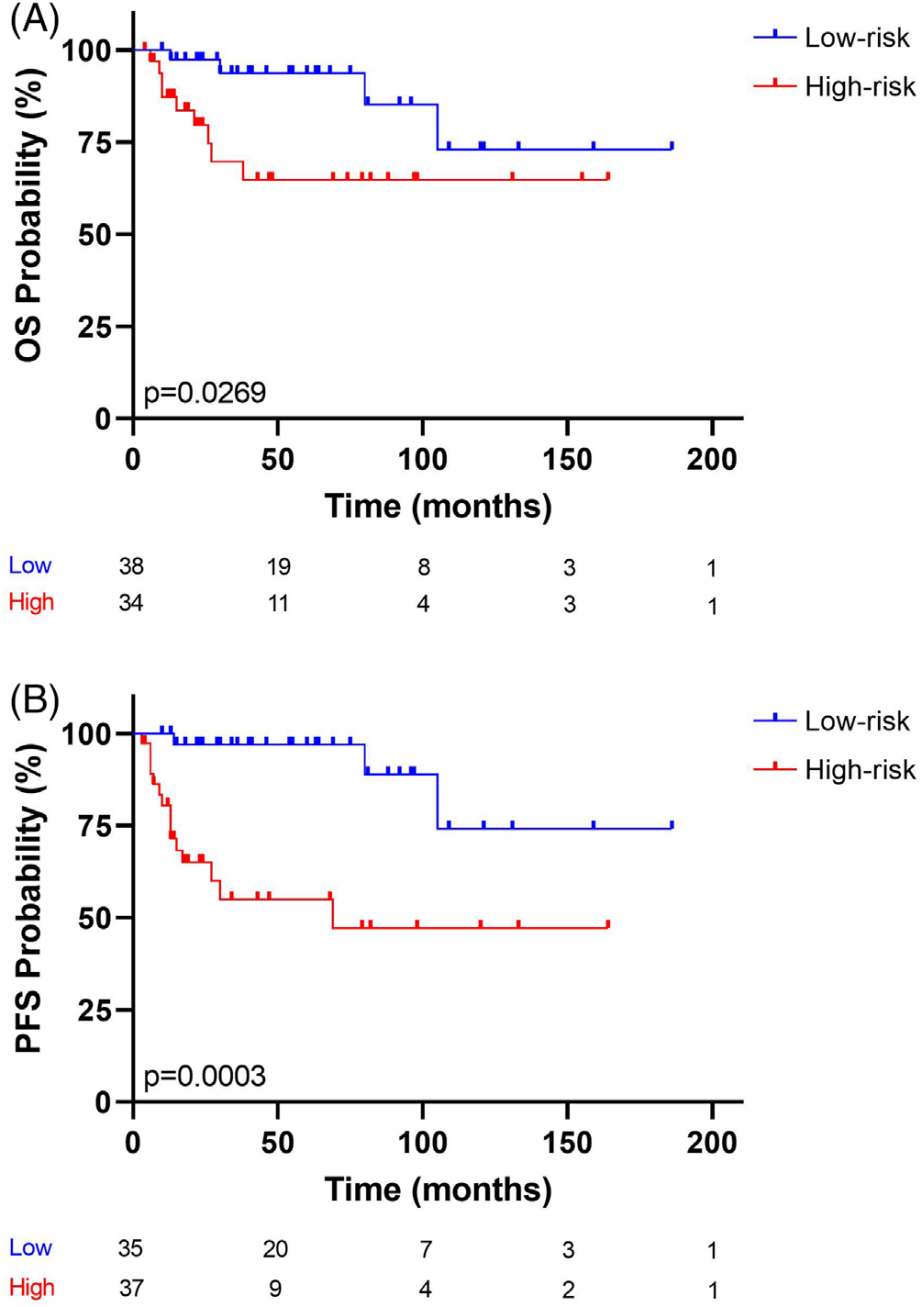
Kaplan–Meier analysis curve of (A) OS status and (B) PFS status. The risk‐prediction model was applied to patients with localized clinical stages (stages I–II). Two risk groups were observed: the blue and red lines represent low‐ and high‐risk, respectively. The vertical bar represents OS or PFS probability (%), while the horizontal bar indicates the follow‐up time in months. Patients at risk at the corresponding time are shown. Probabilities are those associated with a log‐rank test

Next, we examined whether this risk model could be applied to localized clinical stages (stages I–II) (72 patients). For OS, the risk score was split into low‐risk (38 patients) and high‐risk (34 patients) groups, using the median as the cut‐point. For PFS, low‐risk (35 patients) and high‐risk (37 patients) groups were compared. Figure 6A shows that the 5‐year OS of the high‐risk group was 64.7%, and that of the low‐risk group was 93.7%. Figure 6B shows that the 5‐year PFS of the high‐risk group was 54.9%, and that of the low‐risk group was 96.9%. The log‐rank analysis revealed significant differences in OS (Figure 6A, *p* = 0.0269) and highly significant differences in PFS (Figure 6B, *p* = 0.0003).

Taken together, these findings indicate that the prognostic model, which combines IPI with both COO and *MYC/BCL2* double expression, determined by NanoString, yields a score that makes it possible to recognize substantive differences in survival probability. This can be used for young patients (for PFS) and patients at advanced (stages III–IV) and localized (stages I–II) clinical stages of the disease.

### 3.4 Validation of the prognostic model in an independent DLBCL series

To evaluate the performance of the predictive model, data from a retrospective validation series of FFPE samples from 166 DLBCL patients (enrolled in two multicenter clinical trials registered as RHDS0305 and DLCL04) were analyzed after they had been treated with immunochemotherapy [31, 32, 33]. These current trials are investigating the potential role of first‐line autologous stem cell transplant consolidation in intermediate‐ or high‐risk DLBCL. Therefore, the population for the validation series was divided into two risk groups.

The characteristics of the validation set are summarized in Supporting information Table S3. The series included 166 cases diagnosed with DLBCL treated with R‐CHOP, with a median follow‐up of 63.8 months (range: 0.3–119). COO analysis divided the series into 98 (59%) GC‐DLBCL, 37 (22.3%) ABC‐DLBCL, and 31 (18.7%) unclassified DLBCL cases. IPI divided the series into 122 (73.5%) intermediate‐risk and 44 (26.5%) high‐risk samples.

The risk model was then applied to the validation series. As a result, the risk score was further split into terciles for OS: low‐risk (58 patients), intermediate‐risk (58 patients), and high‐risk (50 patients) groups. For PFS, low‐risk (58 patients), intermediate‐risk (52 patients), and high‐risk (56 patients) groups were established.

**FIGURE 7:**
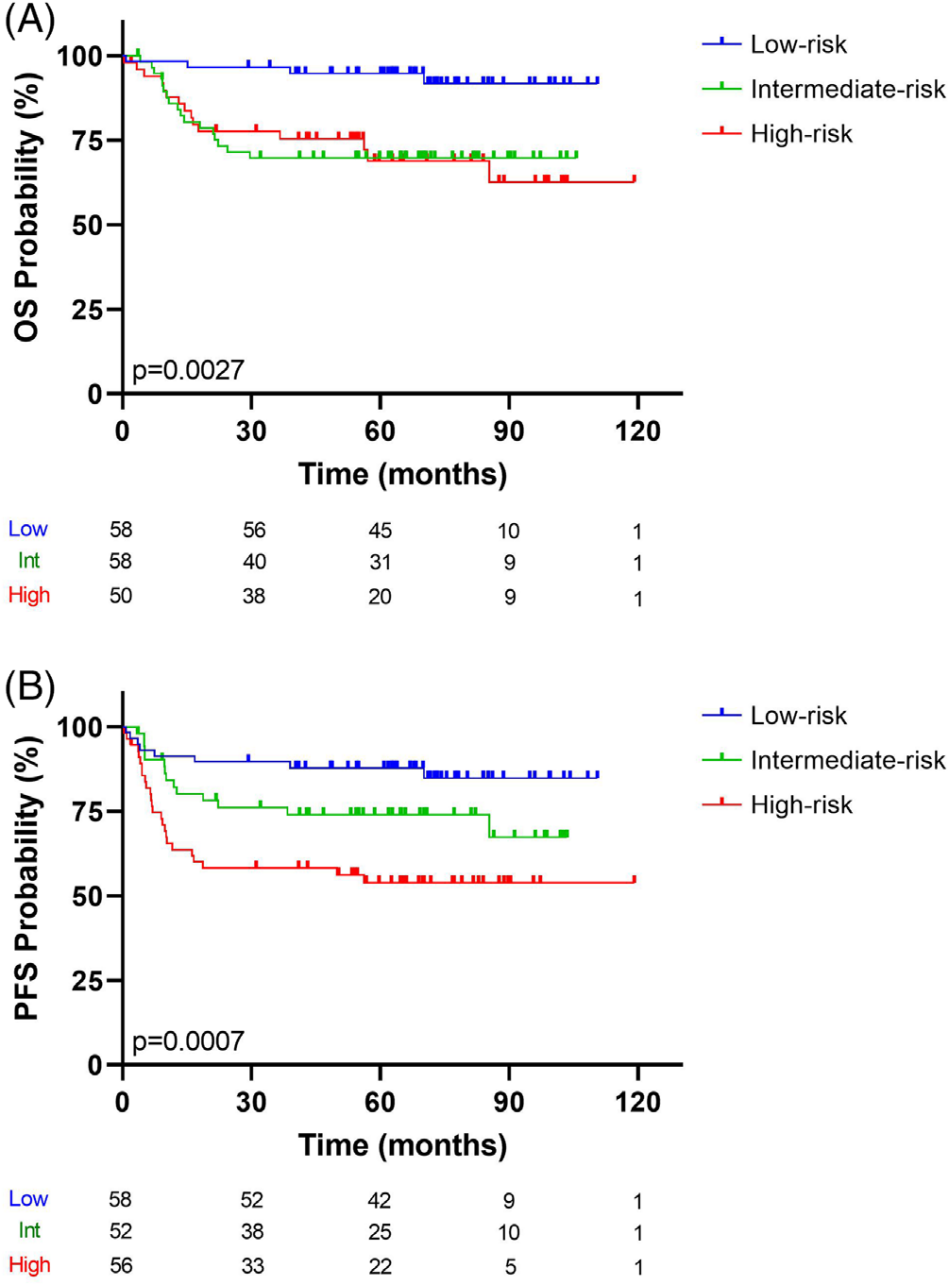
Kaplan–Meier analysis for (A) OS status and (B) PFS status. The risk‐prediction model was applied in an independent series for validation. Three groups were observed: blue, green, and red lines represent low‐, intermediate‐, and high‐risk, respectively. The vertical bar represents OS or PFS probability (%), while the horizontal bar indicates the follow‐up time in months. Patients at risk at the corresponding time are shown. Probabilities are those associated with a log‐rank test

Figure 7A shows that the 5‐year OS of the high‐risk group was 69%, and that of the low‐risk group was 94.8%. Figure 7B shows that the 5‐year PFS of the high‐risk group was 54%, and that of the low‐risk group was 87.9%. The differences between groups were statistically significant (low‐risk vs. intermediate‐risk vs. high‐risk groups, *p* < 0.005) (Figure 7A and B for OS and PFS, respectively).

OS probabilities for the three groups were: low‐risk, HR = 0.2866, 95% CI: 0.1473–0.5575; intermediate‐risk, HR = 3.85, 95% CI: 1.28–11.60 (*p* = 0.0167); and high‐risk, HR = 7.32, 95% CI: 2.4–21.8 (*p* = 0.000345).  Analysis of PFS identified the following HRs: low‐risk, HR = 0.4004, 95% CI: 0.2240–0.7155; intermediate‐risk, HR = 3.07, 95% CI: 1.30–7.24 (*p* = 0.0105); and high‐risk, HR = 5.63, 95% CI: 2.52–12.60 (*p* = 0.00002). Risk scores for each patient in the validation series are presented in Supporting information Table S9. The Cox analysis demonstrated that the intermediate‐ and high‐risk groups were associated with poorer OS and PFS. Overall, we report robust findings that validated the prognostic model in an independent DLBCL series.

## 4 DISCUSSION

Although R‐CHOP continues to be the most common treatment for DLBCL patients, there are no standardized ways of predicting lack of response or progression after treatment, which makes it more difficult to offer therapeutic alternatives to these patients. Here, we demonstrate the value and reproducibility of an integrative risk model to predict PFS and OS in a large DLBCL series homogeneously treated with R‐CHOP. We employed a customized 27‐gene panel for GEP using NanoString because of the applicability of this technique in paraffin‐embedded samples. The design of the panel included genes that provide information about COO, and the expression of *MYC/BCL2* and other genes already known to predict OS and PFS in DLBCL cases [11, 20, 22, 24, 29, 38–42]. Cases were selected carefully, excluding those with mediastinal large B‐cell lymphoma, a lymphoma type with similar morphology but entirely different biology and prognosis. Results were validated in an independent DLBCL series.

We do not intend for this prognostic model to capture the full range of the genetic and epigenetic changes responsible for the diversity of DLBCLs. COO was proposed almost 20 years ago[43] as a bridge between the biology of germinal center differentiation in normal B cells and the DLBCL prognosis. Although the COO signatures capture essential parts of DLBCL pathogenesis, new findings are revealing the spectrum of the underlying molecular history of DLBCL to be broader than previously thought [44, 45]. Part of this complexity arises from the deregulation of the expression of *MYC* and *BCL2* genes because of translocations and other genetic events. Although the most robust way of capturing the contribution of *MYC* and *BCL2* to DLBCL pathogenesis is to identify the translocations deregulating their expression, there is solid evidence that a clinically substantive increase in expression of *MYC* and *BCL2* may depend on genetic and epigenetic events that occur in addition to translocations [20, 46].

Our own group and others have published several studies, all of which have concluded that the IHC analysis of COO markers and MYC/BCL2 protein expression could be used to predict the response to R‐CHOP [18, 19, 47–50], or to recognize a group with an increased risk of central nervous system relapse [51]. However, contradictory results have also been obtained [41, 52]. probably arising from the inherent variability of the IHC techniques. This prompted us to perform this study in paraffin‐embedded tissue using a customized NanoString platform in the hope of generating more robust and reproducible results. Those obtained here are consistent with the findings of the German high‐grade lymphoma study for DLBCL patients treated with R‐CHOP [11], even though the foci of the two studies differ. Our approach is also able to recognize a higher proportion of DLBCL cases that fail to respond to R‐CHOP than reported in recent published articles that have identified either a high‐grade DLBCL subclass that is limited to the GCB group [53], or 9% of DLBCL cases with poor prognosis [54]. Nevertheless, the great majority of the cases recognized by the two research groups belong to the category with low survival probability, since the higher levels of expression of *BCL2* and *MYC* genes are commonly revealed by all these three approaches. The results obtained in the current study confirm previous COO data [9] and are quite similar to those recently described by Derenzini and coworkers. They combined *BCL2/MYC* and *NFKBIA*, a surrogate marker of NFKB activity [55], and confirmed the clinical applicability of GEP techniques to DLBCL patient stratification and the value of combining markers measuring *BCL2/MYC* expression with others recognizing the contribution of COO (our model) or NF‐kB activity (Derenzini's model) [55].

This study is not comparable with those of Ennishi et al. ADDIN EN.CITE or Sha et al [54]. The former study focused on identifying a subset (27%) of DLBCL‐GC type cases with aggressive behavior, while we have generated a model that can be applied to GC and ABC subtypes, recognizing a tercile of very high‐risk DLBCL cases. Sha and coworkers proposed a GEP classifier that recognizes 9% of DLBCL cases (most with a GC phenotype) and a more aggressive behavior [54]. Comparison of the genes used in these three studies suggests that cases recognized by Ennishi and Sha as being molecularly high‐grade fall into the tercile of cases with the greatest aggressivity, as seen in our study [53, 54]. This study has selected the COO Nanostring signature as a consistent and reproducible way of capturing COO [43, 56], rather some individual genes of the COO signature like LMO2 or others [57]. This study has also been restricted to genes capturing DLBCL complexity, but we have excluded genes expressed by the microenvironment, an additional layer of complexity that would require a specific further study [31, 57, 58].

This proposal combines a consolidated prognostic clinical model with the main sources of biological variation among the DLBCL cases—COO and *MYC/BCL2* expression—in a risk model that produces a patient‐specific risk estimate based on a firmly established molecular approach that can be used with paraffin‐embedded tissues. The model is also applicable to early stages and younger patients, which is something that models based on IHC techniques have failed to achieve [37].

This study was performed using consecutive routine cases diagnosed with DLBCL, and thereby closely mimicked the routine composition of cases that receive this diagnosis.

An obvious question that arises is whether this can not only stratify patients into different risk groups, but also help establish a different therapeutic approach. To obtain an answer will require specific clinical trials to be designed and executed, although here we have demonstrated that the risk score can be applied to patients of different ages and clinical stages, encouraging the pursuit of risk‐adjusted therapeutic schemes. Our study was not designed to identify specific therapeutic targets for these high‐risk patients, but other experimental studies have shown that MYC and BCL2 can be targeted using BET and BCL2 inhibitors [59].

## CONFLICT OF INTEREST

M.A.P. declares having received lecture fees and advisory board fees from Millennium/Takeda, Jansen, NanoString, Kyowa Kirin, Gilead, and Celgene.

The authors declare that they have no significant relationships with, or financial interests, in any commercial companies pertaining to this article.

## Supporting information

Supporting information.

Supporting information.

Supporting information.

## Data Availability

The authors uploaded the array data to BioProject (Reference will be included). For original data, please contact marta.rodriguezm@quironsalud.es.

## References

[1] Swerdlow SH , Campo E , Pileri SA , Harris NL , Stein H , Siebert R , et al. The 2016 revision of the World Health Organization classification of lymphoid neoplasms. Blood 2016;127(20):2375‐90. 26980727 10.1182/blood-2016-01-643569 PMC4874220

[2] Sujobert P , Salles G , Bachy E . Molecular classification of diffuse large B‐cell lymphoma: what iss clinically relevant? Hematology/Oncology Clinics 2016;30(6):1163‐77. 10.1016/j.hoc.2016.07.001 27888873

[3] Alizadeh AA , Eisen MB , Davis RE , Ma C , Lossos IS , Rosenwald A , et al. Distinct types of diffuse large B‐cell lymphoma identified by gene expression profiling. Nature 2000;403(6769):503‐11. 10676951 10.1038/35000501

[4] Wright G , Tan B , Rosenwald A , Hurt EH , Wiestner A , Staudt LM . A gene expression‐based method to diagnose clinically distinct subgroups of diffuse large B cell lymphoma. Proc Natl Acad Sci U S A. 2003;100(17):9991‐96. 12900505 10.1073/pnas.1732008100 PMC187912

[5] Lenz G , Wright GW , Emre NC , Kohlhammer H , Dave SS , Davis RE , et al. Molecular subtypes of diffuse large B‐cell lymphoma arise by distinct genetic pathways. Proc Natl Acad Sci U S A 2008;105(36):13520‐5. 18765795 10.1073/pnas.0804295105 PMC2533222

[6] Rosenwald A , Wright G , Chan WC , Connors JM , Campo E , Fisher RI , et al. The use of molecular profiling to predict survival after chemotherapy for diffuse large‐B‐cell lymphoma. N Engl J Med 2002;346(25):1937‐47. 12075054 10.1056/NEJMoa012914

[7] Xu J , Liu JL , Medeiros LJ , Huang W , Khoury JD , McDonnell TJ , et al. MYC rearrangement and MYC/BCL2 double expression but not cell‐of‐origin predict prognosis in R‐CHOP treated diffuse large B‐cell lymphoma. Eur J Haematol 2020;104(4):336‐43. 31944390 10.1111/ejh.13384

[8] Abdulla M , Hollander P , Pandzic T , Ednersson SB , Andersson PO , Hultdin M , et al. Cell‐of‐origin determined by both gene expression profiling and immunohistochemistry is the strongest predictor of survival in patients with diffuse large B‐cell lymphoma. Am J Hematol 2020;95(1):57‐67. 31659781 10.1002/ajh.25666 PMC6916573

[9] Scott DW , Mottok A , Ennishi D , Wright GW , Farinha P , Ben‐Neriah S , et al. Prognostic significance of diffuse large B‐cell lymphoma cell of origin determined by digital gene expression in formalin‐fixed paraffin‐embedded tissue biopsies. J Clin Oncol 2015;33(26):2848‐56. 26240231 10.1200/JCO.2014.60.2383 PMC4554747

[10] Nowakowski GS , Feldman T , Rimsza LM , Westin JR , Witzig TE , Zinzani PL . Integrating precision medicine through evaluation of cell of origin in treatment planning for diffuse large B‐cell lymphoma. Blood Cancer J 2019;9(6):48. 31097684 10.1038/s41408-019-0208-6 PMC6522601

[11] Staiger AM , Ziepert M , Horn H , Scott DW , Barth TFE , Bernd HW , et al. Clinical impact of the cell‐of‐origin classification and the MYC/BCL2 dual expresser status in diffuse large B‐cell lymphoma treated within prospective clinical trials of the german high‐grade non‐Hodgkin's lymphoma study group. J Clin Oncol 2017;35(22):2515‐26. 28525305 10.1200/JCO.2016.70.3660

[12] Lee J , Hue SS , Ko SQ , Tan SY , Liu X , Girard LP , et al. Clinical impact of the cell‐of‐origin classification based on immunohistochemistry criteria and Lymph2Cx of diffuse large B‐cell lymphoma patients in a South‐east Asian population: a single center experience and review of the literature. Expert Rev Hematol. 2019;12(12):1095‐105. 31592693 10.1080/17474086.2019.1677152

[13] Younes A , Sehn LH , Johnson P , Zinzani PL , Hong X , Zhu J , et al. Randomized phase III trial of ibrutinib and rituximab plus cyclophosphamide, doxorubicin, vincristine, and prednisone in non‐germinal center B‐cell diffuse large B‐cell lymphoma. J Clin Oncol 2019;37(15):1285‐95. 30901302 10.1200/JCO.18.02403 PMC6553835

[14] Nowakowski GS , Chiappella A , Witzig TE , et al. ROBUST: Lenalidomide‐R‐CHOP versus placebo‐R‐CHOP in previously untreated ABC‐type diffuse large B‐cell lymphoma. Future Oncol 2016;12(13):1553‐63. 27089170 10.2217/fon-2016-0130 PMC5551933

[15] Davies A , Cummin TE , Barrans S , Maishman T , Mamot C , Novak U , et al. Gene‐expression profiling of bortezomib added to standard chemoimmunotherapy for diffuse large B‐cell lymphoma (REMoDL‐B): an open‐label, randomised, phase 3 trial. Lancet Oncol 2019;20(5):649‐62. 30948276 10.1016/S1470-2045(18)30935-5 PMC6494978

[16] Saez AI , Saez AJ , Artiga MJ , Pérez‐Rosado A , Camacho FI , Díez A , et al. Building an outcome predictor model for diffuse large B‐cell lymphoma. Am J Pathol 2004;164(2):613‐22. 14742266 10.1016/S0002-9440(10)63150-1 PMC1602255

[17] Sanchez E , Chacon I , Plaza MM , Muñoz E , Cruz MA , Martinez B , et al. Clinical outcome in diffuse large B‐cell lymphoma is dependent on the relationship between different cell‐cycle regulator proteins. J Clin Oncol 1998;16(5):1931‐9. 9586912 10.1200/JCO.1998.16.5.1931

[18] Batlle‐Lopez A , Gonzalez de Villambrosia S , Francisco M , Malatxeberria S , Sáez A , Montalban C , et al. Stratifying diffuse large B‐cell lymphoma patients treated with chemoimmunotherapy: GCB/non‐GCB by immunohistochemistry is still a robust and feasible marker. Oncotarget 2016;7(14):18036‐49. 26910115 10.18632/oncotarget.7495 PMC4951269

[19] Hu S , Xu‐Monette ZY , Tzankov A , Green T , Wu L , Balasubramanyam A , et al. MYC/BCL2 protein coexpression contributes to the inferior survival of activated B‐cell subtype of diffuse large B‐cell lymphoma and demonstrates high‐risk gene expression signatures: a report from The International DLBCL Rituximab‐CHOP Consortium Program. Blood 2013;121(20):4021‐31; quiz 250. 23449635 10.1182/blood-2012-10-460063 PMC3709650

[20] Rosenwald A , Bens S , Advani R , Barrans S , Copie‐Bergman C , Elsensohn MH , et al. Prognostic significance of MYC rearrangement and translocation partner in diffuse large B‐cell lymphoma: a study by the Lunenburg lymphoma biomarker consortium. J Clin Oncol 2019;37(35):3359‐68. 31498031 10.1200/JCO.19.00743

[21] Scott DW , King RL , Staiger AM , Ben‐Neriah S , Jiang A , Horn H , et al. High‐grade B‐cell lymphoma with MYC and BCL2 and/or BCL6 rearrangements with diffuse large B‐cell lymphoma morphology. Blood 2018;131(18):2060‐64. 29475959 10.1182/blood-2017-12-820605 PMC6158813

[22] Johnson NA , Slack GW , Savage KJ , Connors JM , Ben‐Neriah S , Rogic S , et al. Concurrent expression of MYC and BCL2 in diffuse large B‐cell lymphoma treated with rituximab plus cyclophosphamide, doxorubicin, vincristine, and prednisone. J Clin Oncol 2012;30(28):3452‐9. 22851565 10.1200/JCO.2011.41.0985 PMC3454768

[23] Beltran BE , Castro D , Paredes S , Miranda RN , Castillo JJ . EBV‐positive diffuse large B‐cell lymphoma, not otherwise specified: 2020 update on diagnosis, risk‐stratification and management. Am J Hematol 2020;95(4):435–45. 32072672 10.1002/ajh.25760

[24] Xu‐Monette ZY , Tu M , Jabbar KJ , Cao X , Tzankov A , Visco C , et al. Clinical and biological significance of de novo CD5+ diffuse large B‐cell lymphoma in Western countries. Oncotarget 2015;6(8):5615‐33. 25760242 10.18632/oncotarget.3479 PMC4467390

[25] Ok CY , Li L , Xu‐Monette ZY , Visco C , Tzankov A , Manyam GC , et al. Prevalence and clinical implications of epstein‐barr virus infection in de novo diffuse large B‐cell lymphoma in Western countries. Clin Cancer Res 2014;20(9):2338‐49. 24583797 10.1158/1078-0432.CCR-13-3157 PMC4014309

[26] Montes‐Moreno S , Odqvist L , Diaz‐Perez JA , Lopez AB , de Villambrosía SG , Mazorra F , et al. EBV‐positive diffuse large B‐cell lymphoma of the elderly is an aggressive post‐germinal center B‐cell neoplasm characterized by prominent nuclear factor‐kB activation. Mod Pathol 2012;25(7):968‐82. 22538516 10.1038/modpathol.2012.52

[27] Menter T , Bodmer‐Haecki A , Dirnhofer S , Tzankov A . Evaluation of the diagnostic and prognostic value of PDL1 expression in Hodgkin and B‐cell lymphomas. Hum Pathol 2016;54:17‐24. 27045512 10.1016/j.humpath.2016.03.005

[28] Gravelle P , Burroni B , Pericart S , Rossi C , Bezombes C , Tosolini M , et al. Mechanisms of PD‐1/PD‐L1 expression and prognostic relevance in non‐Hodgkin lymphoma: a summary of immunohistochemical studies. Oncotarget 2017;8(27):44960‐75. 28402953 10.18632/oncotarget.16680 PMC5546533

[29] Hu S , Xu‐Monette ZY , Balasubramanyam A , Manyam GC , Visco C , Tzankov A , et al. CD30 expression defines a novel subgroup of diffuse large B‐cell lymphoma with favorable prognosis and distinct gene expression signature: a report from the International DLBCL Rituximab‐CHOP Consortium Program Study. Blood 2013;121(14):2715‐24. 23343832 10.1182/blood-2012-10-461848 PMC3700465

[30] International Non‐Hodgkin's Lymphoma Prognostic Factors P . A predictive model for aggressive non‐Hodgkin's lymphoma. N Engl J Med 1993;329(14):987‐94. 8141877 10.1056/NEJM199309303291402

[31] Ciavarella S , Vegliante MC , Fabbri M , De Summa S , Melle F , Motta G , et al. Dissection of DLBCL microenvironment provides a gene expression‐based predictor of survival applicable to formalin‐fixed paraffin‐embedded tissue. Ann Oncol 2018;29(12):2363‐70. 30307529 10.1093/annonc/mdy450 PMC6311951

[32] Cortelazzo S , Tarella C , Gianni AM , Ladetto M , Barbui AM , Rossi A , et al. Randomized trial comparing R‐CHOP versus high‐dose sequential chemotherapy in high‐risk patients with diffuse large b‐cell lymphomas. J Clin Oncol 2016;34(33):4015‐22. 28199143 10.1200/JCO.2016.67.2980

[33] Chiappella A , Martelli M , Angelucci E , Brusamolino E , Evangelista A , Carella AM , et al. Rituximab‐dose‐dense chemotherapy with or without high‐dose chemotherapy plus autologous stem‐cell transplantation in high‐risk diffuse large B‐cell lymphoma (DLCL04): final results of a multicentre, open‐label, randomised, controlled, phase 3 study. Lancet Oncol 2017;18(8):1076‐88. 28668386 10.1016/S1470-2045(17)30444-8

[34] Scott DW , Wright GW , Williams PM , Lih CJ , Walsh W , Jaffe ES , et al. Determining cell‐of‐origin subtypes of diffuse large B‐cell lymphoma using gene expression in formalin‐fixed paraffin‐embedded tissue. Blood 2014;123(8):1214‐7. 24398326 10.1182/blood-2013-11-536433 PMC3931191

[35] Hans CP , Weisenburger DD , Greiner TC , Gascoyne RD , Delabie J , Ott G , et al. Confirmation of the molecular classification of diffuse large B‐cell lymphoma by immunohistochemistry using a tissue microarray. Blood 2004;103(1):275‐82. 14504078 10.1182/blood-2003-05-1545

[36] Gonzalez‐Barca E , Coronado M , Martin A , Montalbán C , Montes‐Moreno S , Panizo C , et al. Spanish Lymphoma Group (GELTAMO) guidelines for the diagnosis, staging, treatment, and follow‐up of diffuse large B‐cell lymphoma. Oncotarget 2018;9(64):32383‐99. 30190794 10.18632/oncotarget.25892 PMC6122355

[37] Barraclough A , Alzahrani M , Ettrup MS , Bishton M , van Vliet C , Farinha P , et al. COO and MYC/BCL2 status do not predict outcome among patients with stage I/II DLBCL: a retrospective multicenter study. Blood Adv 2019;3(13):2013‐21. 31285189 10.1182/bloodadvances.2019000251 PMC6616265

[38] Young KH , Leroy K , Moller MB , Colleoni GWB , Sánchez‐Beato M , Kerbauy FR , et al. Structural profiles of TP53 gene mutations predict clinical outcome in diffuse large B‐cell lymphoma: an international collaborative study. Blood 2008;112(8):3088‐98. 18559976 10.1182/blood-2008-01-129783 PMC2569165

[39] Ennishi D , Takeuchi K , Yokoyama M , Asai H , Mishima Y , Terui Y , et al. CD5 expression is potentially predictive of poor outcome among biomarkers in patients with diffuse large B‐cell lymphoma receiving rituximab plus CHOP therapy. Ann Oncol 2008;19(11):1921‐6. 18573805 10.1093/annonc/mdn392

[40] Rimsza LM , Leblanc ML , Unger JM , Miller TP , Grogan TM , Persky DO , et al. Gene expression predicts overall survival in paraffin‐embedded tissues of diffuse large B‐cell lymphoma treated with R‐CHOP. Blood 2008;112(8):3425‐33. 18544678 10.1182/blood-2008-02-137372 PMC4467875

[41] Salles G , de Jong D , Xie W , Rosenwald A , Chhanabhai M , Gaulard P , et al. Prognostic significance of immunohistochemical biomarkers in diffuse large B‐cell lymphoma: a study from the Lunenburg Lymphoma Biomarker Consortium. Blood 2011;117(26):7070‐8. 21536860 10.1182/blood-2011-04-345256

[42] Horn H , Ziepert M , Becher C , Barth TFE , Bernd HW , Feller AC , et al. MYC status in concert with BCL2 and BCL6 expression predicts outcome in diffuse large B‐cell lymphoma. Blood 2013;121(12):2253‐63. 23335369 10.1182/blood-2012-06-435842

[43] Rosenwald A , Wright G , Chan WC , Connors JM , Campo E , Fisher RI , et al. The use of molecular profiling to predict survival after chemotherapy for diffuse large‐B‐cell lymphoma. New Engl J Med 2002;346(25):1937‐47. 12075054 10.1056/NEJMoa012914

[44] Chapuy B , Stewart C , Dunford AJ , Kim J , Kamburov A , Redd RA , et al. Molecular subtypes of diffuse large B cell lymphoma are associated with distinct pathogenic mechanisms and outcomes. Nat Med 2018;24(5):679‐90. 29713087 10.1038/s41591-018-0016-8 PMC6613387

[45] Schmitz R , Wright GW , Huang DW , Johnson CA , Phelan JD , Wang JQ , et al. Genetics and pathogenesis of diffuse large B‐cell lymphoma. N Engl J Med 2018;378(15):1396‐407. 29641966 10.1056/NEJMoa1801445 PMC6010183

[46] Ziepert M , Lazzi S , Santi R , Vergoni F , Granai M , Mancini V , et al. A 70% cut‐off for MYC protein expression in diffuse large B cell lymphoma identifies a high‐risk group of patients. Haematologica 2020;105(11):2667–70. 33131258 10.3324/haematol.2019.235556 PMC7604633

[47] Visco C , Li Y , Xu‐Monette ZY , Miranda RN , Green TM , Li Y , et al. Comprehensive gene expression profiling and immunohistochemical studies support application of immunophenotypic algorithm for molecular subtype classification in diffuse large B‐cell lymphoma: a report from the International DLBCL Rituximab‐CHOP Consortium Program Study. Leukemia 2012;26(9):2103‐13. 22437443 10.1038/leu.2012.83 PMC3637886

[48] Meyer PN , Fu K , Greiner TC , Smith LM , Delabie J , Gascoyne RD , et al. Immunohistochemical methods for predicting cell of origin and survival in patients with diffuse large B‐cell lymphoma treated with rituximab. J Clin Oncol 2011;29(2):200‐7. 21135273 10.1200/JCO.2010.30.0368 PMC3058275

[49] Iqbal J , Meyer PN , Smith LM , Johnson NA , Vose JM , Greiner TC , et al. BCL2 predicts survival in germinal center B‐cell‐like diffuse large B‐cell lymphoma treated with CHOP‐like therapy and rituximab. Clin Cancer Res 2011;17(24):7785‐95. 21933893 10.1158/1078-0432.CCR-11-0267 PMC7394278

[50] Choi WW , Weisenburger DD , Greiner TC , Piris MA , Banham AH , Delabie J , et al. A new immunostain algorithm classifies diffuse large B‐cell lymphoma into molecular subtypes with high accuracy. Clin Cancer Res 2009;15(17):5494‐502. 19706817 10.1158/1078-0432.CCR-09-0113 PMC7289055

[51] Savage KJ , Slack GW , Mottok A , Sehn LH , Villa D , Kansara R , et al. Impact of dual expression of MYC and BCL2 by immunohistochemistry on the risk of CNS relapse in DLBCL. Blood 2016;127(18):2182‐8. 26834242 10.1182/blood-2015-10-676700

[52] Gutierrez‐Garcia G , Cardesa‐Salzmann T , Climent F , González‐Barca E , Mercadal S , Mate JL , et al. Gene‐expression profiling and not immunophenotypic algorithms predicts prognosis in patients with diffuse large B‐cell lymphoma treated with immunochemotherapy. Blood 2011;117(18):4836‐43. 21441466 10.1182/blood-2010-12-322362

[53] Ennishi D , Jiang A , Boyle M , Collinge B , Grande BM , Ben‐Neriah S , et al. Double‐hit gene expression signature defines a distinct subgroup of germinal center B‐cell‐like diffuse large b‐cell lymphoma. J Clin Oncol 2019;37(3):190‐201. 30523716 10.1200/JCO.18.01583 PMC6804880

[54] Sha C , Barrans S , Cucco F , Bentley MA , Care MA , Cummin T , et al. Molecular high‐grade B‐cell lymphoma: defining a poor‐risk group that requires different approaches to therapy. J Clin Oncol 2019;37(3):202‐12. 30523719 10.1200/JCO.18.01314 PMC6338391

[55] Derenzini E , Mazzara S , Melle F , Motta G , Fabbri M , Bruna R , et al. A 3‐gene signature based on MYC, BCL‐2 and NFKBIA improves risk stratification in diffuse large B‐cell lymphoma. Haematologica 2021;106:2405‐2416. 32817282 10.3324/haematol.2019.236455 PMC8409021

[56] Scott DW , Wright GW , Williams PM , Lih CJ , Walsh W , Jaffe ES , et al. Determining cell‐of‐origin subtypes of diffuse large B‐cell lymphoma using gene expression in formalin‐fixed paraffin‐embedded tissue. Blood, J Am Soc Hematol 2014;123(8):1214‐17. 10.1182/blood-2013-11-536433 PMC3931191 24398326

[57] Alizadeh AA , Gentles AJ , Alencar AJ , Liu CL , Kohrt HE , Houot R , et al. Prediction of survival in diffuse large B‐cell lymphoma based on the expression of 2 genes reflecting tumor and microenvironment. Blood 2011;118(5):1350‐8. 21670469 10.1182/blood-2011-03-345272 PMC3152499

[58] Croci GA , Au‐Yeung RKH , Reinke S , Staiger AM , Koch K , Oschlies I , et al. SPARC‐positive macrophages are the superior prognostic factor in the microenvironment of diffuse large B‐cell lymphoma and independent of MYC rearrangement and double‐/triple‐hit status. Ann Oncol 2021;32(11):1400‐09. 34438040 10.1016/j.annonc.2021.08.1991

[59] Deng M , Xu‐Monette ZY , Pham LV , Wang X , Tzankov A , Fang X , et al. Aggressive B‐cell lymphoma with MYC/TP53 dual alterations displays distinct clinicopathobiological features and response to novel targeted agents. Mol Cancer Res. 2021. 19:249‐260. 33154093 10.1158/1541-7786.MCR-20-0466 PMC8092941

