# Supplementary material for: An integrated prognostic model for diffuse large B‐cell lymphoma treated with immunochemotherapy
Source: EJHaem. 2022 May 3;3(3):722–33. doi: 10.1002/jha2.457 (PMC9422037; doi:10.1002/jha2.457)
Supplement: Supplementary file 1 — Supporting information. [file JHA2-3-722-s003.doc]

**SUPPORTING INFORMATION**

**Supplemental figure legends**


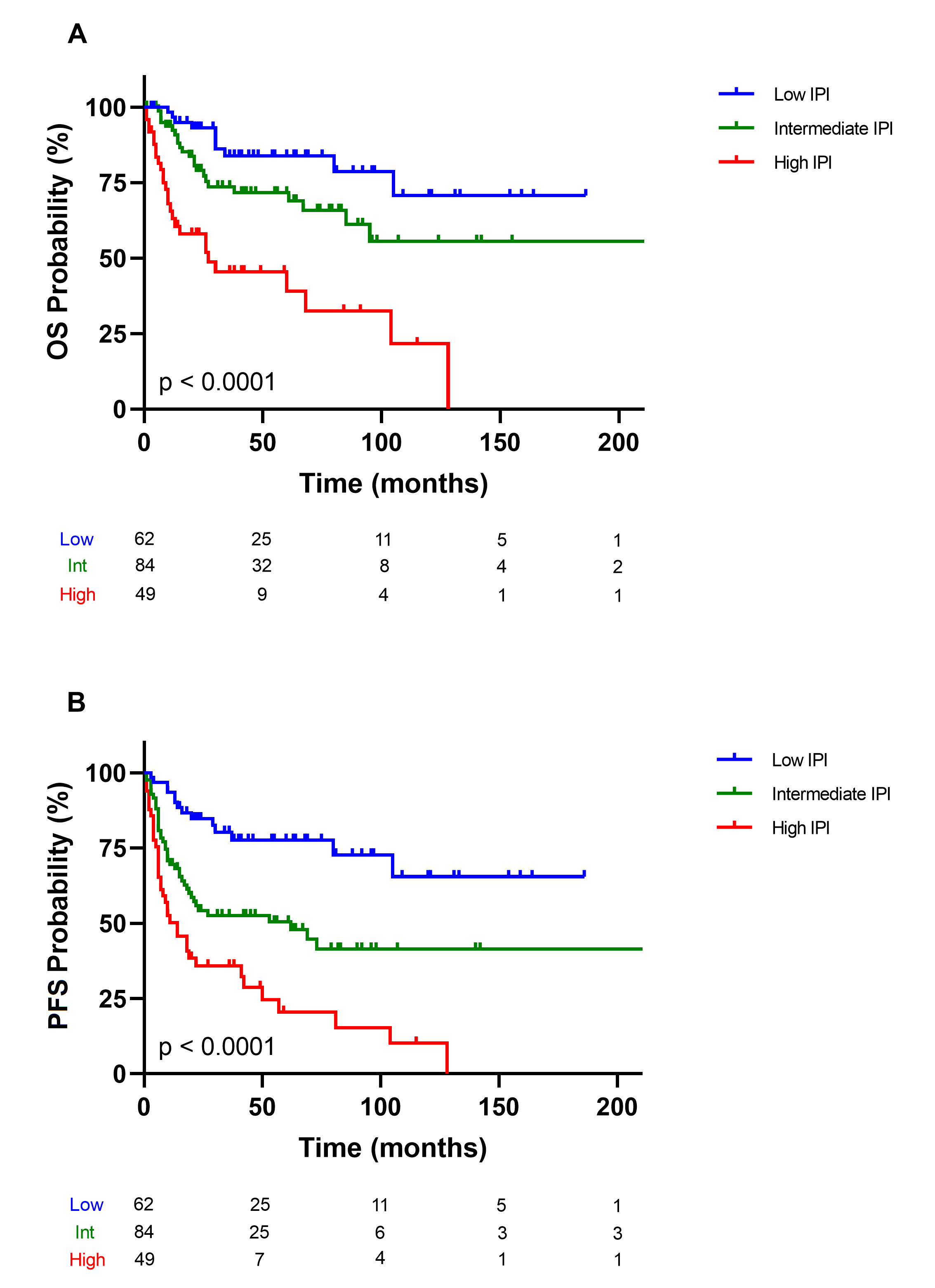
**Supplemental Figure 1. Kaplan-Meier analysis of IPI for A) OS status and B) PFS status.** Blue line represents low IPI cases; green line represents intermediate IPI cases; red line represents high IPI cases. The vertical bar represents the OS and the PFS probability (%), while the horizontal bar represents the follow-up time in months. Patients at risk at the corresponding time point are shown. *P-value* was calculated by Log-rank test.

**Supplemental Figure 2. Kaplan-Meier analysis of COO for A) OS status and B) PFS status.** Blue line represents GC group; green line represents ABC group; red line represents Unclassified group. The vertical bar represents the OS and the PFS probability (%), while the horizontal bar represents the follow-up time in months. Patients at risk at the corresponding time point are shown. *P-value* was calculated by Log-rank test.

**
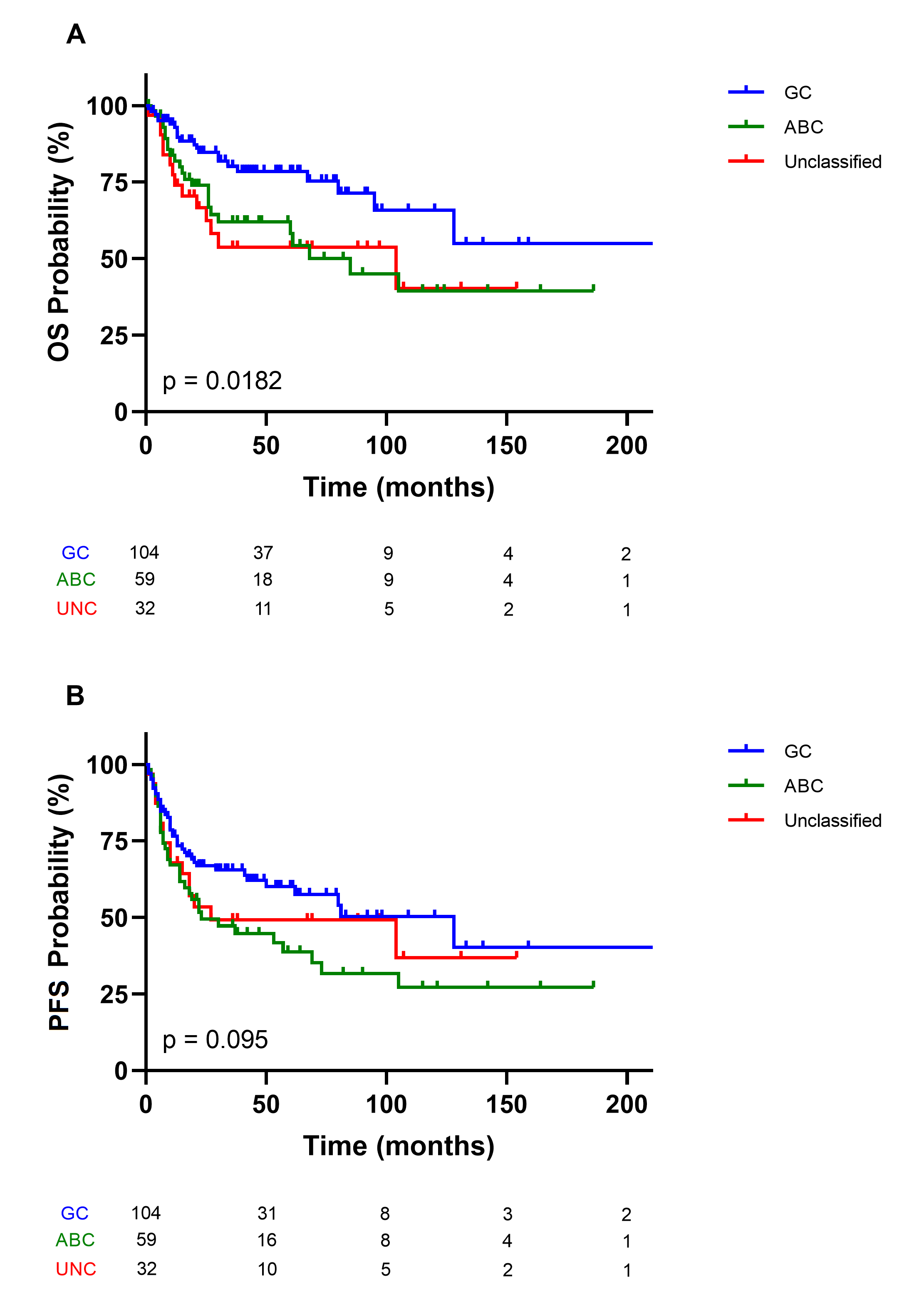
**

**Supplemental Figure 3. Kaplan-Meier analysis of *BCL2/MYC* for A) OS status and B) PFS status.** Blue line represents *BCL2/MYC* low expression; green line represents *BCL2/MYC* high expression. The vertical bar represents the OS and the PFS probability (%), while the horizontal bar represents the follow-up time in months. Patients at risk at the corresponding time point are shown. *P-value* was calculated by Log-rank test.


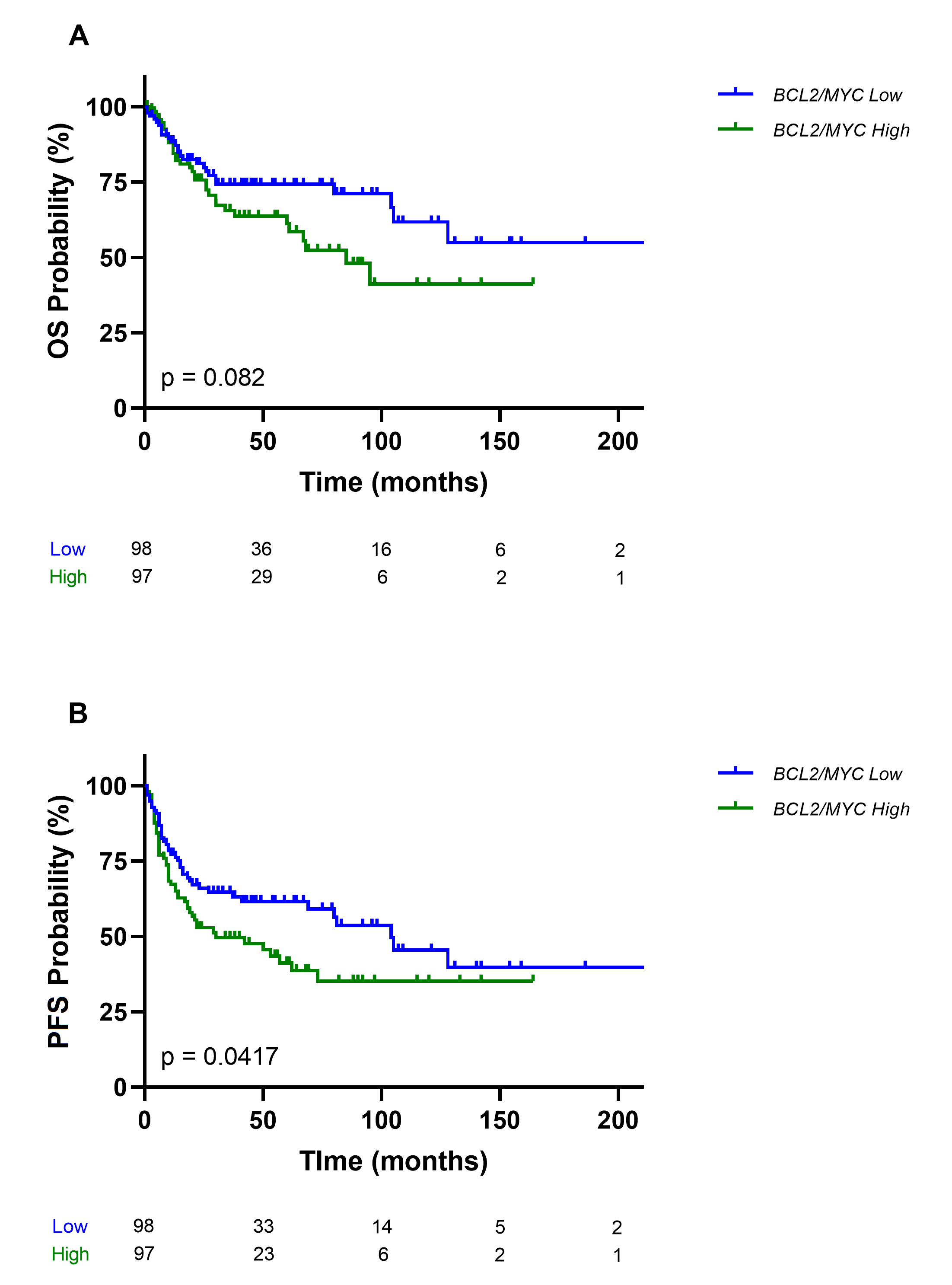


**Supplemental Figure 4. Bar graph showing patients categorized by COO when the model was applied.** The vertical bar represents low-, intermediate- and high-risk groups, while the horizontal bar represents the number of DLBCL samples. Bars are grouped by position for risk groups of one categorical variable, with color indicating the secondary category level within each group (GC, ABC and “unclassified”). *P-value* was calculated by Fisher's exact test.

**
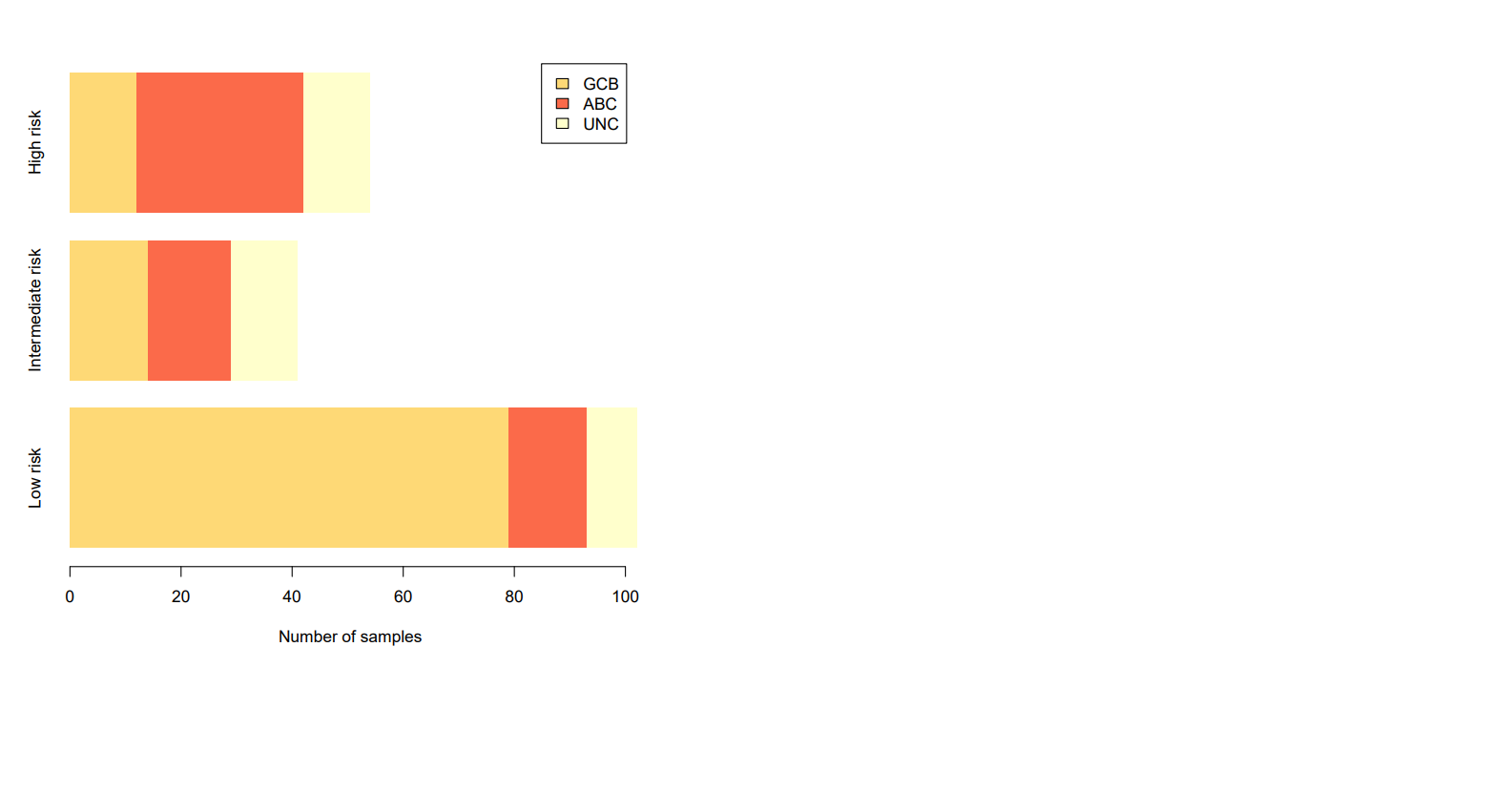
**

**Supplemental table legends**

**Supplemental Table 1. Main clinical features of the Diffuse Large B-Cell Lymphoma series (N=197).** ECOG PS, Eastern Cooperative Oncology Group performance status; IPI, International Prognostic Index; LDH, lactate dehydrogenase; NA, not available; ULN, upper level of normal.

**Supplemental Table 2. Main molecular features of the Diffuse Large B-Cell Lymphoma series (N=197).** COO, cell-of-origin; GC, germinal center B-cell; ABC, activated B-cell; IHC, immunohistochemistry; TR, translocations; NA, not available.

**Supplemental Table 3. Main clinical and molecular features of the Diffuse Large B-Cell Lymphoma validation series (N=166).** COO, cell-of-origin; GC, germinal center B-cell; ABC, activated B-cell; IPI, International Prognostic Index.

**Supplemental Table 4. List of customized 27-gene panel.** A set of 5 housekeeping genes was used to normalize the gene expression values.

| **Genes** | | | **Housekeeping genes** |
| --- | --- | --- | --- |
| *ASB13* | | *MME* | *ISY1* |
| *BCL2* | | *MYBL1* | *R3HDM1* |
| *CCDC50* | | *MYC* | *TRIM56* |
| *CD5* | | *PDL1* | *UBXN4* |
| *CREB3L2* | | *PIM2* | *WDR55* |
| *CYB5R2* | | *RAB7L1* |  |
| *IRF4* | | *S1PR2* |  |
| *ITPKB* | | *SERPINA9* |  |
| *LIMD1* | | *TNFRSF13B* |  |
| *MAML3* | | *TNFRSF8* |  |
| *MKI67* | | *TP53* |  |
|  |  | | |

**Supplemental Table 5. Antibodies used in the study. Clone and source.**

| **MARKER** | **CLONE** | **COMMERCIAL FIRM** | **REFERENCE** | **CONTROL** |
| --- | --- | --- | --- | --- |
| CD10 | 56C6 | DAKO-AGILENT | M7308 | TONSIL |
| BCL6 | PG-B6p | DAKO-AGILENT | M7211 | TONSIL |
| MUM1 | MUM1p | DAKO-AGILENT | M7259 | TONSIL |
| P53 | DO-7 | DAKO-AGILENT | M7001 | TONSIL |
| CD5 | 4C7 | DAKO-AGILENT | IR082 | TONSIL |
| CD30 | Ber-H2 | DAKO-AGILENT | M0751 | TONSIL |
| Ki67 | MIB-1 | DAKO-AGILENT | GA506 | TONSIL |
| PD-L1 | 22C3 | DAKO-AGILENT | SK006 | TONSIL |
| BCL2 | 124 | DAKO-AGILENT | M0887 | TONSIL |
| MYC | Y69 | VENTANA-ROCHE | 790-4628 | TONSIL |
| EBV | EBER-1 | VENTANA-ROCHE | 800–2842 | TONSIL |
| CYCLIN D1 | EP12 | DAKO-AGILENT | M3642 | TONSIL |
| CD20 | L26 | DAKO-AGILENT | M0755 | TONSIL |

**Supplemental Table 6. Univariate and multivariate analyses using Cox proportional-hazards models for OS status.** Hazard ratios (HRs) from univariate and multivariate Cox analysis of these variables: *BCL2, CD5, MKi67, MYC, PDL1, TNFRSF8, TP53*, COO, and double expression of *MYC/BCL2* by NanoString gene-expression analysis and IPI score. Error bars indicate the 95% confidence interval of the HR.

**Supplemental Table 7. Univariate and multivariate analyses using Cox proportional-hazards models for PFS status.** Hazard ratios (HRs) from univariate and multivariate Cox analysis of these variables: *BCL2, CD5, MKi67, MYC, PDL1, TNFRSF8, TP53*, COO, and double expression of *MYC/BCL2* by NanoString gene-expression analysis and IPI score. Error bars indicate the 95% confidence interval of the HR.

**Supplemental Table 8. The risk scores for each sample generated by the model, in the discovery series.**

Additional excel file that is provided separately.

**Supplemental Table 9. The risk scores for each sample generated by the model, in the validation series.**

Additional excel file that is provided separately.
